# The ways specialist nursing students understand the work in the ambulance service - a national Swedish phenomenographic study

**DOI:** 10.1080/17482631.2022.2099023

**Published:** 2022-07-07

**Authors:** Kim Wallin, Anders Bremer, Bengt Fridlund, Ulrica Hörberg, Carina Werkander Harstäde

**Affiliations:** a Centre of Interprofessional Cooperation within Emergency Care (CICE), Linnaeus University, Vaxjo, Sweden; b Faculty of Health and Life Sciences, Linnaeus University, Vaxjo, Sweden

**Keywords:** Ambulance service, clinical studies, education, learning, phenomenography, specialist nurses, work role transition

## Abstract

**Objective:**

To explore and describe the ways specialist ambulance nursing (SAN) students understand the work in the ambulance service.

**Design, sample, and measurements:**

An explorative descriptive design was carried out through individual interviews with 16 SAN students from all parts of Sweden and analysed in accordance with the phenomenographic tradition.

**Findings:**

Five different ways of understanding the work were described and each was assigned a metaphor; The medical role; The practical role; The patient-oriented role; The commanding role; and The comprehensive role. Several aspects concerning personal, organizational, and situational conditions affecting the understanding and the distribution of these roles in the specific care assignment were identified and presented in a hierarchical model of the outcome space.

**Conclusions:**

This study contributes with a new perspective on supporting role clarity for registered nurses (RN) working in the ambulance service (AS). Specialization and experiential learning are needed to support an understanding of all aspects of the work in order to develop a professional competence aligned with the challenges faced in the AS. The development of expertise in the AS needs a contextualized understanding rooted in a theoretical framework that addresses a holistic perspective towards patients’ needs.

## Introduction

Work role transitions, including specialization, for registered nurses (RN) are described as personally, professionally, and emotionally challenging. This socialization process involves endeavouring to attain a new professional self and clinical know-how (Arrowsmith et al., 2016). The search for a new professional identity is influenced by processes of self-awareness, role-identification, and the contextualizing of a new practice (Rasmussen et al., 2018). Educational interventions that support role clarity and contextual awareness are thus of great importance for facilitating work role transitions (Yardley et al., 2018), for reducing feelings of anxiety, stress, and a sense of being in a “no man’s land” (Arrowsmith et al., 2016). Role clarity and the ways health care clinicians understand their work affects both their way of performing it and how they strive to develop new competence (Dall’Alba & Sandberg, 2006). However, work role transitions should not be viewed as one moment in time, but rather as a continuum of lifelong professional development (Yardley et al., 2018).

Educators should apply educational interventions facilitating the understanding of the professional role, the clinical context, and the objects of learning when supporting RNs in work role transitions. Educational strategies within health care education have, in line with learning theories (Illeris, 2009), shifted the focus from a teacher-centred to a learner-centred approach (Aliakbari et al., 2015). This development has placed the learner in focus and addresses an educational design that challenges the learners’ actual understanding of their future professional role and clinical context. Previous research focusing on the education system for ambulance service (AS) clinicians in the UK and Australia (Devenish et al., 2016) has shown that the students have stereotypical preconceptions of their future role when entering the paramedic programme. These preconceptions are influenced by e.g., media and television, gender, social class, individual schooling and education, and the parental level of education and type of employment. Educational interventions and clinical practice were identified as important for supporting the development of “road-readiness”, role clarity, and a professional socialization. Similar research exploring RNs specializing in ambulance care and their understanding of the professional role are scant.

RNs have different pathways for specialization within nursing depending on the educational system in their country (Dury et al., 2014). Sweden is one of a few European countries where the university-trained RN (3-year bachelor degree) is believed to have the most suitable basic competence for working in the AS (Wihlborg et al., 2014). The one-year specialist programme within ambulance care for RNs in Sweden is at the master level, containing 10 weeks of clinical practice. Supporting Swedish RNs transition to become a specialized ambulance nurse (SAN) is problematic due to several aspects concerning role clarity. Firstly, inconsistencies exist in several countries, Sweden included, concerning opinions about the educational design, the desired competence and what constitutes the professional role for clinicians working in the AS (Jensen, 2020; Plummer et al., 2017; Wihlborg et al., 2014). This latter concerns an expanding and changing role, responsibility, and scope of practice for AS clinicians relating to a change in societal expectations and demands placed on the AS. Secondly, the clinicians themselves describe a multifaceted, changing, and unclear professional role (Rosén et al., 2018), with research indicating varying understanding of nursing objectives in the Swedish AS workforce (Forsell et al., 2020). Thirdly, the educational content and design vary between the nine Swedish universities providing the ambulance programme, thus indicating varying preparations for the SAN students prior to clinical practice (Sjölin et al., 2015). Fourthly, SANs supporting these specialist students during clinical practice report of challenges concerning varying needs, relating to differences in knowledge, attitudes, and occupational background when entering the clinical practice (Wallin et al., 2020).

In summary, the complex and somewhat unclear educational environment for the support of the understanding of SAN students concerning the professional role and clinical context during specialist training needs clarification. It could be assumed that the students’ understanding of their future work differs from those of the experienced AS clinicians, and one way of illustrating strategies for supporting the work role transition and understanding of SAN students is by an explorative phenomenographic approach as described by Larsson and Holmström (2007). A phenomenographic exploration generates a description of the professional role from the students’ qualitatively different ways of understanding and thus serves as a basis for supporting a learner-centred approach. Such findings are useful in discussions concerning the role objectives and supporting a developed understanding among both students and experienced clinicians. Are the views of the professionals aligned with the students’ understanding of the work? Do the SAN students bring knowledge and understanding from their preparatory university studies that are aligned with the challenges confronted with in the AS? Are there other aspects that affect their development of understanding of the professional role during clinical practice in the AS? In order to address these queries, the aim of the study was to explore and describe the ways specialist ambulance nursing students understand the work in the ambulance service.

## Materials and methods

### Design and method description

An explorative and descriptive design was carried out through individual interviews with 16 SAN students. The data was analysed in accordance with the phenomenographic tradition, aiming to describe and understand *how* people perceive the world (Marton, 1981). Phenomenography, with its roots in educational research, has been applied in health care research since the 1990s (Sjöström & Dahlgren, 2002). The intention with a phenomenographic study is to explore and describe the variations in people’s ways of understanding a phenomenon, and not directed at the phenomenon as such. There is thus a distinction in phenomenography between the world as it is, and the world as it is perceived by people. The former perspective is labelled the first order perspective (the what), and the latter is labelled the second-order perspective (the how), which also is the target in a phenomenographic study (Larsson & Holmström, 2007).

### Educational and clinical context

University trained RNs, including specialized RNs, have become the majority in the Swedish AS workforce during the last two decades (Wallin et al., 2021). This is in line with Swedish legislation demanding that the advanced life-support ambulance units are manned with at least one RN to be able to administer drugs. In addition, several of the 21 publicly funded health care regions demand specialized RNs for employment in the AS. The Swedish AS consisted of 53% specialized RNs (dominated by SANs), 28% RNs, and 19% emergency medical technicians in 2021 (Table I). The specialist programme in ambulance care for RNs in Sweden contains 40 weeks of fulltime studies in both theoretical and practical courses. The University Chancellor’s Office stipulates a national curriculum with 12 overall degree objectives applied to all Swedish specialist nursing programmes, accompanied by two additional degree objectives that are specific for each specialist focus. These generally formulated degree objectives are interpreted independently by each university into the course objectives and course content of the syllabus in the professional programmes. There is no detailed legal definition of what constitutes the professional role of SANs, and there is thus room for interpretation when syllabi are developed at each university. The Swedish National Association for Ambulance Nurses and the Swedish Society of Nursing developed recommendations for the desired competence of Swedish SANs as a response to these prerequisites (RAS—The Swedish Association of Ambulance Nurses and SSF—the Swedish Society of Nursing, 2012/2022). However, these recommendations have no legal obligations for the development of the content or design of the specialist programmes. All but three of the nine universities demand that the RNs have worked full time as a RN for 0.5–3 years when applying for specialist training in 2021. Since there is no national legislation demanding specialist-trained RNs for employment within the Swedish AS, some RNs have already worked in the AS when entering their specialist training. All programmes implement clinical practice within the AS, during which SAN students work as a third team member of an ordinary ambulance crew. The students are assessed in collaboration between their AS preceptor and a university teacher in line with the universities’ course objectives during these clinical weeks.

**Table I.** Educational background and profession of fulltime ambulance clinicians in the Swedish ambulance service (year 2021) in the 21 healthcare regions.

Healthcare region*	Specialized RNs n (%)	Registered nurses (RN) n (%)	Emergency medical technicians n (%)	Total, n
Kronoberg	116 (78.5)	23 (15.5)	9 (6)	148
Gotland	25 (78)	1 (3)	6 (19)	32
Halland	134 (74)	28 (16)	18 (10)	180
Västernorrland	142 (69)	64 (31)	0 (0)	206
Jämtland Härjedalen	80 (60)	36 (27)	17 (13)	133
Jönköping	127 (58)	62 (28)	31 (14)	220
Kalmar	133 (58)	64 (28)	33 (14)	230
Skåne	374 (58)	89 (14)	185 (28)	648
Uppsala	107 (56)	41 (21)	44 (23)	192
Västra Götaland	485 (56)	222 (26)	153 (18)	860
Blekinge	55 (54)	37 (36)	10 (10)	102
Stockholm	510 (52)	183 (18.5)	291 (29.5)	984
Norrbotten	116 (51.5)	34 (15)	75 (33.5)	225
Östergötland	104 (51)	68 (33)	32 (16)	204
Värmland	131 (47)	118 (42)	31 (11)	280
Örebro	121 (46)	107 (41)	34 (13)	262
Dalarna	135 (45)	124 (42)	39 (13)	298
Sörmland	72 (44)	65 (39)	28 (17)	165
Västerbotten	69 (41)	58 (35)	40 (24)	167
Västmanland	54 (29)	88 (47)	45 (24)	187
Gävleborg	56 (22)	169 (67)	27 (11)	252
**All regions in total**	**3146 (53)**	**1681 (28)**	**1148 (19)**	**5975**

*Ranked from the highest to the lowest proportion of specialized RNs.

### Participants

There were 218 SAN students admitted to the nine Swedish universities providing the specialist programme for RNs in ambulance care from the autumn of 2019 to the autumn of 2020. Forty-five of these (38 females and 7 males) were identified as not having any prior experiences of working in the AS. These were all invited to participate in the study with up to five emails being sent. This resulted in 16 students agreeing to participate (Table II). At least one participant came from each of the nine universities, and their clinical practice was performed at twelve ambulance departments in all parts of Sweden. Thirteen students were females, and the ages ranged from 26 to 57 years (mean 34.8). Their experiences as a RN prior to commencing the specialist programme were from one to ten years (mean 4.3), and the medical field in which they had previously worked as an RN varied. The students’ experiences of AS clinical practice when being interviewed ranged from one to seven weeks (mean 3.1).

**Table II.** Socio-demographic and professional characteristics of the specialist ambulance nursing students (*n = 16*).

Participant	University	Ambulance departement	Gender (age)	Years as RN	RNs’ previous workplace*	Weeks of clinical practice when interviewed
1	A	1	F(32)	4	PSY+PC	5
2	B	2	F(28)	1	ICU+H	5
3	C	3	F(42)	6	PC	5
4	D	4	F(32)	8	PC	4
5	D	6	F(35)	2	H+ PC	7
6	E	5	F(34)	10	PC	6
7	F	7	M(30)	4	H+ PC+PSY	2
8	G	3	F(26)	2	H	3
9	H	8	F(57)	7	PSY+H+ ED	4
10	H	9	M(29)	3	H+ ED	1
11	H	8	F(47)	3	H	1
12	H	8	F(42)	7	H+ PC	1
13	H	11	F(30)	4	ED+H+ PC	1
14	H	8	F(26)	2	ED+PC	2
15	I	12	F(38)	4	H+ PC	2
16	I	10	M(29)	2	ED	1

* PC = primary care (out-of-hospital nursing), H = hospital ward (various), ED = emergency department, PSY = psychiatric care, ICU = intensive care unit.

### Data collection

Written permission to invite and interview students were procured from the heads of departments at the students’ universities. The data were collected in semi-structured individual interviews from March until November 2020. The first author performed all the interviews, using Zoom-meetings, in which all the participants were asked four main questions following the phenomenographic approach of Larsson and Holmström (2007): *What is the core of your professional work in the ambulance service?, When have you been successful in your work?, What is difficult, or what hinders you in your work*?, and *What is your most important task?* These were followed with more in-depth questions such as: *“Can you tell me more about … ?”* and *“What do you mean by … ?”* to gain richer descriptions of their experiences. The participants were asked repeatedly to provide examples from their own experiences to avoid descriptions of opinions, attitudes, values, and thoughts (Larsson & Holmström, 2007). The interviews lasted between 16 and 62 minutes with a mean time of 35 minutes. The total time for all the interviews were 555 minutes, generating 238 transcribed pages. All the interviews were electronically recorded and transcribed verbatim by a professional agency.

### Ethical considerations

The study followed the principles of the Declaration of Helsinki and the Swedish Ethical Review Authority approved of the study (No. 2019–03157). The participants were informed about their rights of withdrawing at any time and were ensured confidentiality. Verbal consent to participate in the study was obtained from the participants and recorded prior to the interviews.

### Data analysis

The data analysis was carried out in accordance with the phenomenographic tradition as described by Larsson and Holmström (2007). The first author read the whole text of all the interviews four times in the initial phase (familiarization), and then marked where the students gave answers to the four main interview questions. This process was repeated five times to safeguard that the marked text was correlated correctly with the whole interview. All the authors then compared the marked text with the main questions. Comparisons and contrasting between the whole interview and the marked text was performed by all the authors in two of the interviews. The first author then looked in the marked text for *what* the focus of the students’ attention was and then *how* he/she described his/her way of working. A preliminary description of each student’s predominant way of understanding the work was made. All the authors then engaged in critical reflections concerning the identified dominant ways of understanding.

The first author then grouped the descriptions into categories, based on similarities and differences. The categories of description were formulated in the next step, and then critically reflected, and discussed among all the authors. The first author then searched the data for non-dominant ways of understanding and created an overview of dominant and non-dominant ways of understanding (Table III). The next step entailed the first author developing a preliminary structure of the outcome space. This was performed by repeatedly going back and forth between the marked sections in the interviews, the dominant and non-dominant ways of understanding, and the categories of description. This process was critically reviewed by all the authors in three meetings in a ten-week period. The validation process of the whole analysis continued until consensus was reached between all the authors. A metaphor was then assigned to each category of description and supporting quotes inserted in agreement among all the authors. Finally, a collegial review of the manuscript was performed by doctoral students and other researchers to critically address the analysis and the presentation of findings.

**Table III.** Overview of the dominant (xx) and non-dominant (x) ways of understanding the work in the ambulance service by specialist ambulance nursing students.

Participant	The medical role (A)	The practical role (B)	The patient-oriented role (C)	The commanding role (D)	The comprehensive role (E)
1	x	x	x		xx
2	x	x	xx	x	
3	x	x	x		xx
4	x	x	x	xx	x
5	x	x	xx		
6	x	x	xx		
7	xx	x	x		
8	x		x	xx	x
9	x	x	xx	x	
10	x		x	x	xx
11	x		x	x	xx
12	xx		x	x	x
13	x	x	x	xx	x
14	x	x	xx		x
15	x	x	xx		x
16	xx	x			

## Findings

**Figure 1.**
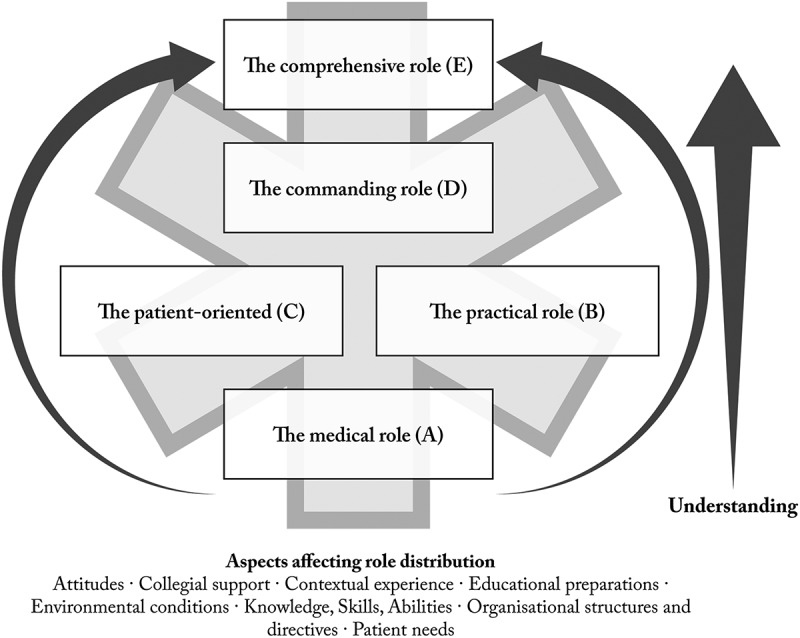
Analytic model of findings—The five roles (A-E) for understanding the work in the ambulance service are linked together in a hierarchical relationship portraying an increasing complexity in the understanding.

There were five qualitatively different ways of understanding the work in the ambulance service; *The medical role* (A); *The practical role* (B); *The patient-oriented role* (C); *The commanding role* (D); and *The comprehensive role* (E). These five descriptive categories, presented below with supporting quotes from the interviews, were finally analytically described in an outcome space (Figure 1).

### The medical role

The work was understood as the responsibility for knowledge and skills in medical assessments and actions for maintaining the patient’s life and physical well-being. Uncertainty about the patient’s previous medical history, lack of support in the medical responsibility, and the varying needs in each care meeting posed a challenge in the role. The medical responsibility for the patient’s bodily functions and survival was dealt with by adhering to rigid templates and guidelines in order to maintain a patient-safe care.

Especially as a newcomer, it’s important to follow the systems quite strictly so as not to miss anything. That is, you work with big C and A to E, that you report according to SBAR and that you always think in your head: “I work according to SAMPLE and ONSET. I ask these important questions, how, when, where”. You know, work systematically all the time. Because if you’ve been there a little longer, yes I absolutely believe that you work according to system, but more “automatically” if you understand. It’s second nature that shows itself. But for us there is no … or for me there is no second nature yet (14).

Focus was on saving lives and relieving suffering from a biomedical perspective, where a structured medical assessment was the basis for succeeding in care. The ability to collect measurement values, clinically assess bodily vital functions, and initiate correct medical treatment was understood as fundamental. Guidelines and structure created security and clarity in the work, where initiated medical treatment and directly measurable findings on the patient’s physical well-being and reduced bodily suffering were correlated to a successful outcome.

We went through the routines that we have regarding hip tracks and yes, when we arrived at the patient, then we just followed the routines that we have in the ambulance to the letter. I did the drugs and the treatments; it gave good results and gave the patient less pain and suffering and the whole care chain worked. Yes, from home to the hospital. Yes, it followed like the textbook example this time (7).

### The practical role

The work was to solve practical challenges, that cannot always be written down in manuals or guidelines, to create a good and safe working environment. The role meant deciding on what should be done to optimize the pace of care, gain access to the right equipment and continually adapt to the environmental challenges that occur. The task was to create good conditions for care at home, in public environments, in changing weather, inaccessible environment, and handling varying safety risks by adapting the technical and practical equipment at hand.

So many transports, I’ve not thought that it takes so much of the time … there are no elevators, there are narrow stairs, it does not have to be in the woods, it can be in the middle of a shopping center, with a lot of people there, there’s very little space. In other words, it’s often the place where the patient is prepared in a good way that takes a lot of time, which creates frustration. And it doesn’t require anything medical, how you say it, but purely practical thinking. You have to be very good at thinking practically (6).

The focus was on managing technical equipment and practical challenges in the prehospital environment. Based on ingenuity and experience-based situated practical knowledge, the purpose was to identify the best solution for geographically reaching the patient, physically conducting care on site, and transferring the patient to a care facility. Good knowledge of working methods, routines, directives, and the ability to work together as a team to deal with the assignments effectively was a priority. The task was to adapt and deal with the practical challenges faced with in each care assignment and maintain assignment objectives as safely and efficiently for the patient as possible during care.

Actually, I mean everything. I don’t know why I just focused on the car, but I really mean everything. How to talk in Rakel (the radio communication equipment). How to report to the emergency room. How to document. You know, everything, everything, everything. So, the whole care encounter. There are situations that are completely new to us as … And it affects how you give the care of course. When you haven’t worked so much with the practical things, it makes you feel insecure when you are out practicing (14).

### The patient-oriented role

The work was based on the patient’s best interests and on the insight into the meaning of one’s own actions for the patient’s feeling of being confirmed, experiencing security and well-being. The role meant performing a thorough assessment and explaining what is done and why, aiming at creating a sense of participation, control and being taken seriously as a patient. Existential and social aspects were incorporated in the assessment and care decisions, where guidelines and directives were adapted to the patient’s needs to provide support in the best way.

Listening to the patient. Why did the patient call, what was the reason for calling from the beginning and what is the real problem as the patient sees it, what is the patient’s biggest problem and how does he feel that he wants help with this and then to adapt oneself based on it (3).

The focus was on the patient’s story by trying to elucidate his/her subjectively perceived care needs, based on a holistic and open-minded view of the human being. Caring actions were focused on continuous information, mutual agreement, and aiming at strengthening the patients’ feelings of security. A constraining of one’s own prejudices and perspectives on the patient’s care needs before and during the care encounter was sought in order to avoid bias in the assessment and provision of care. Verbal and non-verbal cues of the patient’s satisfaction and feelings of security were a measurement of a successful care.

I don’t think it’s that damn difficult, really. It’s just mainly having an open body language, looking at the person and listening to what they say, and then express a willingness to help. It’s not that difficult, because I feel that many people probably go a little over the heads of the patients and come in like a storm and do what they should, as they have already decided that they should do, and based on algorithms, guidelines, and everything that may exist in order to keep your back free, and then you just move on. You don’t listen so much to the patients; you don’t want to do this little extra that often means a lot (10).

### The commanding role

The work was understood as entailing an independent and powerful role, demanding a sense of security in the professional role and flexibility in medical, practical, and communicative abilities when faced with a variety of needs, demands and situations concerning aspects of life, death and the wellbeing of patients and relatives. This required a well-developed interpersonal and educational ability when being forced to convey difficult messages, or being faced with both verbal and nonverbal threats, ethical dilemmas, emotional distress, and existential suffering.

The previous nursing role, there it was the doctors they listened to, (I gave) more information. There you were more of a nursing specialist. Here it’s really that you get … My experience is that patients listen very much to the ambulance staff. But then you also have to make difficult decisions in the ambulance, you didn’t need to do that in the hospital because it’s the doctors who make the difficult decisions and who they get a little angry at. There we were more these kind people. // So, there you always had someone behind you, and it was they who had to convey the difficult decisions. // But in the ambulance, it’s you who says this stuff that “you must not come in”, or “you must not … no, but you should not come in because it is not … you will only be allowed to sit in the emergency room for a long time and then it is better that you apply to your health center because they will not do anything in the emergency room anyway”. So, you must be very flexible, I think, you have to learn that (11).

The focus was on taking control and leading the care assignments when balancing between the patients’ understanding of their perceived health care needs and the professional assessment. This meant trying to establish a professional trust, by educating and arguing for alternatives for care and treatment. Controlling and leading the care encounters also meant being able to take over from the patient or argue for the professional assessment in encounters that can sometimes entail conflicts with relatives or patients.

Communication with the patient, that the patient understands what we do and why we do it … why we make certain decisions. Yes, but precisely this interaction with the patient, that you’re on the same level. Because we have an amazing knowledge when it comes to healthcare and medicine and all that, while the patient doesn’t have it. So, for the patient it may seem like it’s very serious, and she will die and so on, while for us it’s not … we can take it easy, breathe. And it’s precisely in such situations that you get the patient on the same wavelength to … yes, so with communication that you come to a joint decision and can calm the patient (13).

### The comprehensive role

The work included having leadership abilities and cooperative skills, when collaborating with other blue light organizations, different health care agencies and authorities in order to optimize decisions about the patient’s care. The role was to identify the patient’s best interests in the situation, in a synthesis of and negotiation with relatives’ wishes and the organizational guidelines and directives of different care bodies, while simultaneously adapting to varying prerequisites in organizational directives and structures depending on the time of the day.

But after a long dialogue with the home healthcare service, the mobile team and the ambulance and the patient involved, it was great in the end, and we later found out that he now has a coordinated and individual care plan so we know what kind of care he should have the next time (14).

The focus was on guiding the patient to the right level of care and coordinating and balancing between the best interests of the patient, the relatives, the healthcare organization, and the ambulance service. Medical, practical, social, existential, and ethical aspects were interwoven in a holistic assessment to optimize the right decision to meet the patient’s needs. The role was to support the patient’s health process both in the short and the long term, based on experiences and understanding of the healthcare organization. Previous care contacts and future care planning were taken into consideration regarding the current decision that was made.

It’s often that it’s the ambulance nurse who ends up in the position of having to provide guidance. It is a guiding role that you end up in. Many (patients) end up outside the system, then you have to try to prod them right into the system again. So, it’s a very overreaching role, you get to meet a lot of different people and different patients, and try to help them in the way they need. And it’s not always emergency care. It requires having a very broad understanding of how the healthcare service works in order to be able to refer someone right. I still don’t understand all that, so how can I think that an ordinary individual in society should be able to understand it? (10).

### Outcome space

The ways the SAN students understood the work in the ambulance service is analytically described as consisting of five qualitatively different roles A-E (Figure 1). These five approaches are linked together in a hierarchical relationship describing an increasing complexity in the understanding of the work, where roles D and E entail the SAN having mastered the roles A-C. The SAN students described their experiences during the interviews by exemplifying with two, or more, care assignments consisting of varying perspectives on the work. In summary, this suggests that their understanding of the work, and the roles applied at different times, were also dependent on the dynamic nature of the patients’ care needs, and the practical and environmental conditions of each care assignment. The findings also suggest that attitudes, collegial support, organizational structures, and preparatory educational interventions influenced aspects of the ways of understanding, the focus, and the distribution of the roles.

The medical role (A) was understood as the basic task for the work. This perspective was depending on the patient’s current medical needs, the practical conditions (the practical role B), the nature of the collegial support, and the student´s knowledge, abilities, and experiences. The novice found it difficult to focus on other roles if uncertainty in the medical (A) and/or the practical role (B) diverted their attention in the caring. A clearer focus on the patient-oriented role (C) was facilitated by an increased sense of security and ability, and/or relieving support from the colleague. However, more experiences did not automatically generate an increased understanding and focus on a patient-oriented perspective (C), but was also related to individual knowledge, values, and attitudes towards the patient’s perceived needs.

The commanding role (D), which was closely linked to the medical role (A) and the patient-oriented role (C), was hierarchically more complex, consisting of leadership abilities, and based on a sense of security in mastering roles A-C. The commanding role (D) involved an independent leading medical role, perceived as being dissimilar to previous RN roles, where information that was difficult to convey, diverging decisions about care needs and health status, dealing with conflicts, and providing existential support were the task. The commanding role (D) appeared to be affected by an imbalance in perspectives, where the patient risks being offended and feeling reprimanded, instead of being supported, educated, and involved if an anchoring in the patient-oriented (C) perspective was lacking.

The most complex and holistic understanding of the work, the comprehensive role (E), was based on a sense of security in all the other roles and paired with a well-developed understanding of the healthcare organization and other collaborative partners. This experience-based situated competence addressed the patients’ needs and the decisions about care measures in a more comprehensive way. The decision-making process adhered to patient needs over time (care chain) and organizational aspects (needs at individual-group-societal level) in a holistic assessment concerning social, medical, existential, and caring needs. However, the dynamic nature of the comprehensive role (E) was affected by the attitudes of the involved AS team members, and/or other care institutions, and challenged by rigid organizational structures or directives, sometimes creating contradictions between the patient’s needs and organizational needs.

## Discussion

The key findings in this study suggest that the SAN students understand the AS work as demanding an experience-based development of competence to address the patients’ care needs in a more comprehensive way. The work contains multiple roles and perspectives that need to be balanced in relation to the specific needs of the patient and the contextual circumstances at hand when simultaneously striving for dealing with a dynamic healthcare environment. The hierarchical relationships in the study findings (Figure 1) show a continuum of the understanding of the work from a protocol-based and limited approach to a more flexible and holistic approach, portraying a multifaceted competence profile. Professional competence is a complex concept in nursing with a number of definitions (Valizadeh et al., 2019) and there are different ways of theorizing and explaining professional development (Dall’Alba & Sandberg, 2006). Professional competence is “*a complex, relative, context-dependent and variable concept, and combines an individual’s knowledge, skills, attitudes, and logical, scientific, and behavioral attributes, on the basis of which one can perform his roles professionally and independently in a standard manner and have an appropriate clinical judgment in different situations*” (Valizadeh et al., 2019, p. 6). According to the framework of Dall’Alba and Sandberg (2006), a professional development towards expertise is not only dependent on the development of a situated skilful know-how that often comes with experiences but is also dependent of the clinicians’ embodied understanding of the work. This means that the individual´s understanding of the work may be static and limited even though the clinical experiences over time contribute to an increased know-how. The understanding of the full complexity of a specific work is thus fundamental in the development of expertise and a professional competence. The competence considered necessary for a specific group of professionals is influenced by e.g., professional organizations, institutions of higher education and governmental agencies (Eraut, 1998). One way of addressing the findings in the present study is therefore by comparing the students’ understanding of the work with the competence desired according to previous research and the viewpoint of the professionals.

The SAN students’ understanding of the work in the present study is in an overall perspective well aligned with previous European research presenting the views of clinicians, university teachers and students on the desired competence for RNs working in the AS (Jansson et al., 2020; Nilsson et al., 2020; Vázquez-Casares & Vidal, 2020; Wihlborg et al., 2014). Even though the European research presents a more detailed description of competence items, these findings correlate well with the five roles identified in the present study. The main areas of overlapping findings address contextualized knowledge, skills, and abilities in medical assessment and treatment, technical and practical skills, communicative and cooperative skills, clinical judgment, educational and leadership abilities, and abilities in teamwork and collaboration with other actors. These similarities in findings may be interpreted as that the educational preparations prior to clinical practice in Sweden are aligned with the expectations from the professionals and supports the SAN students’ understanding of their future role and clinical context. One possible explanation is that the ambulance specialist programmes in Sweden, in addition to the two generally held specific degree objectives in the national curriculums, implement educational interventions influenced by the competence requirements for SAN´s proposed by the Swedish Society of Nursing and the Swedish National Association for Ambulance Nurses (RAS—The Swedish Association of Ambulance Nurses and SSF—the Swedish Society of Nursing, 2012/2022), which were later developed into the ambulance nurse competence-scale by Nilsson et al. (2020).

However, the findings from this phenomenographic study should be viewed as a map of possible ways of understanding the work (Larsson & Holmström, 2007), and thereby showing the varying levels of understanding the SAN students have. This is supported by previous Swedish research concerning clinical practice in the AS, where SANs report wide variations in the students’ preparedness (Wallin et al., 2020) and the SAN students themselves address feelings of being unprepared for the challenging clinical context of the AS (Axelsson et al., 2016; Bremer & Holmberg, 2020; Wallin et al., 2013). This variance in preparedness and understanding may be related to the fact that the SAN students, when entering their specialist programme, vary in terms of years of work experiences as RNs. It may also be related to a variance in the Swedish ambulance specialist programmes educational design and content prior to clinical practice (Sjölin et al., 2015). Other studies have also indicated that clinical AS experiences for RNs is of the greatest importance for developing a competence in dealing with the challenges faced with in the AS (Hörberg et al., 2017; Jansson et al., 2020; Wihlborg et al., 2017). This is in line with (Vázquez-Casares & Vidal, 2020), who conclude that only eight out of 60 identified competence items for SANs were considered to be part of the basic training for RNs, according to Spanish university professors. The remaining 52 competence items were suggested as demanding post-graduate programmes, complementary training, and experiential learning in the AS to be supported. In summary, and in line with the present study, this indicates that the educational and experiential backgrounds of RNs alone are insufficient in supporting a competence and an understanding of the demands of the work in the AS.

The roles that most clearly related to a need for experiential learning in the present study were the commanding and the comprehensive role. These roles were understood as highly contextual and perceived as being unlike previous RN roles. The medical and patient-oriented roles were understood as more related to previous RN work, even though there were several aspects concerning the sole medical responsibility and the decision-making process that needed adaptation and further training to adhere to the prerequisites of the AS clinical context. However, the practical role was the least mentioned role, which is interesting. The challenging aspects of work in the AS relating to technical and practical conditions have previously been described by both students (Devenish et al., 2016; Wallin et al., 2013) and clinicians (Reay et al., 2018; Wallin et al., 2021). Several of the professionals most desired competence items for Swedish SANs in a study by Wihlborg et al. (2014), were related to technical and practical aspects in the AS work. This may be problematic in relation to the present study where none of the participants understood the practical role in the work as being dominant, and several of the SAN students did not acknowledge it at all. The reason for this is unclear, but analytically conceivable reasons may be that the practical role has mostly been dealt with by the preceptor or his/her colleague, or this role has simply not been clearly defined for the student.

The medical role, and the responsibility for the patient’s biomedical status was understood as the foundational task for the AS work when addressing findings from the present study in a societal perspective. This understanding of the societal role for the AS in a historical perspective is supported by previous research in countries where the AS are staffed with both paramedics (Devenish et al., 2016; Jensen, 2020) and RNs (Suserud, 2005). However, this is a highly simplified and dualistic human perspective on the work and the patient’s needs, which contradicts the development in demands placed on the AS during the last decades. Internationally, the contemporary AS are faced with patient needs and societal changes resulting in a development towards more than just protocol driven emergency medical treatment and an expanding scope of practice (Jensen, 2020; Plummer et al., 2017; Vázquez-Casares & Vidal, 2020). These developments are related to an increasing number of patients (Lowthian et al., 2011) with e.g., non-emergency conditions (Rosén et al., 2018), mental health care needs (Todorova et al., 2022), and older adults with multimorbidity and palliative care needs (Van Vuuren et al., 2021). This has resulted in the need for advanced medical assessments, care measures, and triaging patients to different levels of care and/or decisions concerning the conveyance or the non-conveyance (Lederman et al., 2019). These challenges and patient needs demand a workforce with reflective practitioners who can deal with ethical dilemmas (Bremer & Holmberg, 2020; Shearer et al., 2021) and complex clinical decisions (Andersson et al., 2019; Bennett et al., 2021) that are in the best interest of both the individual patient and the health care organizations’ forced needs of prioritizing care in the best interest of all patients (Kingswell et al., 2017). The need for a development of non-technical skills and a clinical decision-making competence for AS clinicians is evident in the international research (Andersson et al., 2019; Bennett et al., 2021), and this correlates well with the comprehensive role in the present study’s hierarchical map of understanding the AS work.

Most of the SAN students in the present study understood the importance of developing a competence combined from a medical and patient-oriented perspective that adhered to the specific healthcare context of the AS. This is interesting when compared with the findings in the study by Sjölin et al. (2015), showing that the syllabi-content of the Swedish SAN programmes primarily focused on supporting the medical perspective, with only a limited focus on supporting an understanding for a patient-oriented and context-related AS knowledge. These contradictory findings may only be speculated about but may be related to aspects such as developments in the Swedish syllabus content since year 2015, attitudes and culture in each ambulance department providing the clinical practice, as well as in the individual SAN students’ clinical background, weeks in clinical practice, knowledge, attitudes, and values. Furthermore, the present study also addressed barriers for being able to focus on a patient-oriented perspective in care. These barriers related to attitudes towards patient needs of the AS, a lack of support from colleagues in other role objectives and being a novice in the AS environment. This is partially surprising, considering that all the SAN students are RNs when entering their specialist programme, and should according to their clinical experiences and educational degree be competent in a patient-oriented nursing perspective. However, the environmental aspects of the work in the AS and a lack of a sense of security and abilities to perform medical assessments may be an obstacle for SAN students’ ability to focus on and develop a patient-oriented ethical competence in clinical judgment (Wallin et al., 2021). Both the present as well as previous studies (Ahlenius et al., 2017; Forsell et al., 2020) of the Swedish AS identify divergent attitudes towards nursing and the understanding of the work that may hinder a patient-oriented perspective. Diverging perspectives on patient care needs, working methods, and nursing objectives in AS teamwork have previously been described as a potential threat to patient safety (Holmberg et al., 2020). This may be viewed as a classic conflict between different objectives in the AS (Ahl et al., 2005), where cultural and historical prerequisites relating to the understanding of AS work possibly contribute to these struggles in perspective. This complexity is further emphasized by the results from the study by Vázquez-Casares and Vidal (2020), where the Spanish university professors valued a protocol-driven care higher than an individualized care-plan in the AS. One way forward is by addressing these issues in the specialist programmes and the ambulance departments by discussions concerning attitudes towards patient-centeredness and nursing objectives in the AS. To address these queries, a theoretical grounding in a framework like the EXPAND-model (Holmberg, 2021) can support the understanding of AS work. This model illustrates the previously mentioned challenges of addressing patients’ needs in a holistic perspective. Educational interventions supporting a competence and understanding that adhere to the challenges faced with in the AS will be facilitated by using a medical and a patient-oriented nursing perspective that is theoretically elaborated and explained. Providing support for a professional development in the AS that enables the development of expertise, a combination of specialist education, AS experience, and educational interventions supporting a life-long development of understanding the complexities of AS work are thus of greatest importance (Dall’Alba & Sandberg, 2006).

### Methodological issues

This study should address aspects of quality that are philosophically and methodologically congruent with the study aim and design in order to promote scientific rigour in qualitative-designed studies (Caelli et al., 2003). According to Sin (2010), a basic quality criterion for phenomenographic studies is the match between the aim and the design. Findings from a second-order perspective can, in a study of human experiences and ways of understanding complex social phenomena with a non-dualistic approach (Marton, 1981), contribute to the development of e.g., an educational tool for supporting novice clinicians’ understandings of more complex aspects of their work (Larsson & Holmström, 2007). The strategic selection of participants, with an invitation to all eligible SAN students, contributed to the included participants having a wide range of socio-demographic and professional characteristics (Table II), which thus enabled conceptual variations to be maximized (Marton, 1981). According to Larsson and Holmström (2007), experiences from a large number of phenomenographic studies have shown that 20 participants is sufficient to generate significant variations of the understanding of a phenomenon. All the authors in the present study assessed and critically discussed the 16 available interviews and agreed that there was sufficient richness in conceptual variations and depth in the data to proceed with the analysis. The rich descriptions of five different metaphors for working in the AS in the study findings are in line with Larsson and Holmström’s (2007) descriptions of an expected phenomenographic outcome of 2–6 metaphors, thus engendering the trustworthiness of the study findings (Sin, 2010).

All the SAN students were asked the same open-ended questions to reduce the risk of the first author´s influence on the participants expressing their experiences during the interviews (Sin, 2010). The use of multiple open-ended probing questions to elicit greater elaboration of the experiences, as well as the importance of the SAN students expressing their own experiences explicitly connected to specific situations, were communicated to the students prior to and were aimed for in the dialogue during the interviews. This was done to avoid descriptions of opinions, attitudes, values, and thoughts (Larsson & Holmström, 2007). The first author initiated the analysis by listening to all the interviews, made notes, and adjusted the transcripts to be as true as possible to the SAN students’ expressed meanings in order to reduce the risk of misinterpretations due to the fact that the interviews were transcribed by a professional agency. Several critical reflective meetings with all the authors were held to maintain a continued reflexive attitude towards the data in the most challenging phase of the phenomenographic analysis, i.e., when separating the structural aspects (what) from the referential aspects (how) (Marton, 1981).

The authors are all experienced in qualitatively analysed designs and approaches, whereas one had specific experience of conducting phenomenographic studies and was therefore recruited to enhance quality (Sin, 2010). Two of the authors are experienced ambulance clinicians, and thus the important role for the three other authors in questioning their preunderstanding throughout the research process was recognized in the planning and execution of the study. To address issues of researcher and participant bias, the authors ensured that none of the authors were involved in the SAN student’s university courses during the data collection, and all eligible participants were informed about this prior to their decision to participate in the study. A collegial review of the manuscript was performed by researchers and doctoral students not participating in the research process in order to address issues of researcher bias for all the authors. A clear description of the data analysis, a presentation of the findings exemplified with excerpts from the raw data, and a linguistically clear reporting of the findings were aimed to enable readers to judge the study’s credibility, confirmability, and authenticity (Sin, 2010). The clear description of the SAN students’ characteristics and their geographical distribution from all universities and several ambulance departments in Sweden facilitates the transferability of the findings and the readers’ possibility of judging the trustworthiness of the data (Sin, 2010). The transferability of the study findings in an international perspective should be carried out with considerations in terms of the diversities in educational systems and the organization of the AS in different countries.

## Conclusion and implications

This study has shed new light on existing knowledge by adding a hierarchical map of the understanding of AS work by SAN students. This map supports professional organizations, institutions of higher education and governmental agencies in discussions concerning role objectives, educational design, and aspects affecting the professional development for SANs. There is a need for educational interventions and further training based on a theoretically rooted framework that supports the intertwining of a medical and patient-oriented perspective to enforce the SAN students’ development of a competence that addresses all aspects of AS work. Educators and preceptors supporting SAN students’ learning have to be aware of the need to address the specific environmental prerequisites of the AS and can use the present study’s findings in education and supervision to support a developed understanding of all aspects of AS work. Ambulance departments need to address workplace attitudes and the importance of supporting a life-long development of understanding AS work in a more comprehensive way to support a development of expertise for AS clinicians. Educational interventions and other aspects affecting SAN students’ understanding of AS work need to be further investigated.
